# A type-specific nested PCR assay established and applied for investigation of HBV genotype and subgenotype in Chinese patients with chronic HBV infection

**DOI:** 10.1186/1743-422X-9-121

**Published:** 2012-06-19

**Authors:** Jing-Jing Nie, Kui-Xia Sun, Jie Li, Jie Wang, Hui Jin, Ling Wang, Feng-Min Lu, Tong Li, Ling Yan, Jing-Xian Yang, Mi-Shu Sun, Hui Zhuang

**Affiliations:** 1 Department of Microbiology, School of Basic Medical Sciences, Peking University Health Science Center, Beijing, 100191, China

**Keywords:** Genotype, Hepatitis B virus, Nested PCR, Subgenotype, Type-specific nested PCR

## Abstract

**Background:**

Many studies have suggested that hepatitis B virus (HBV) genotypes show not only geographical distribution and race specificity, but also are associated with disease progression and response to interferon treatment. The objective of this study was to develop a nested polymerase chain reaction (nPCR) assay for genotypes A-D and subgenotypes B1, B2, C1 and C2 of hepatitis B virus (HBV) and to investigate the distribution characteristics of HBV genotypes/subgenotype in China.

**Methods:**

After redesigning the primers and optimizing the reaction conditions using common Taq polymerase, the sensitivity, specificity and reproducibility of the method were evaluated using plasmids and serum samples. In total, 642 serum samples from patients with chronic HBV infection were applied to investigate the distribution of HBV genotype and subgenotype in China.

**Results:**

The genotype and subgenotype could be identified when the HBV DNA load of a sample was ≥10^2.3^ IU/mL. For the 639 successfully genotyped samples, the sequencing results of 130 randomly selected samples (20.3%, 130/639) were consistent with those of the nPCR method. The present study showed that HBV genotype B (11.2%, 72/642), C (68.2%, 438/642) and D (7.2%, 46/642) were circulating in China, while genotype C was the dominant strain except for western region where genotype D was the prevalent strain. The main subgenotypes of genotypes B and C were B2 (87.5%, 63/72) and C2 (92.9%, 407/438), respectively.

**Conclusions:**

The low-cost nPCR method would be a useful tool for clinical and epidemiological investigation in the regions where genotypes A-D are predominant.

## Background

An increasing number of studies have suggested that hepatitis B virus (HBV) genotypes show not only geographical distribution and race specificity, but also are associated with disease progression and response to interferon treatment [1,2].

According to sequence divergence of greater than 8% over the entire genome, or greater than 4% for the S gene, HBV has been classified into eight different genotypes A-H [3-6]. Different subgenotypes for each genotype have also been identified and defined by sequence divergence of between 4% and 8% of the full genome [7].

Hepatitis B is endemic in China, with a hepatitis B surface antigen (HBsAg) carrier rate of 7.18% [8]. HBV molecular epidemiological studies showed that genotypes B and C were predominant in China. Genotype A (few and scattered), D (predominant in Uyghur patients of Xinjiang) and C/D recombinant (mainly found in Tibet) were also detected in China [9,10]. The predominant subgenotype of B was B2, and that of C was C2, while B1 and C1 were also present in China [11].

There are several methods for HBV genotyping. Sequence analysis of the entire genome [3] or of the S gene [4] is considered as the gold standard for genotyping. Other methods include type-specific PCR [12-15], restriction fragment length polymorphism assay (RFLP) [16-18], line probe assay [19], and specific monoclonal antibody assay [20,21], etc. Among these methods, the type-specific PCR method has been used widely for clinical and epidemiological surveys due to its rapid detection, low cost, high specificity and sensitivity, and superior results for the diagnosis of mixed infections [22].

Dr. Naito and his colleagues developed a type-specific nPCR method for genotyping of HBV in 2001 [15], and it was considered as a classical method. Following Naito’s method, one round type-specific PCR (multiplex PCR) methods was developed by some scholars [12-14]. Our lab adopted Naito's method for genotyping since 2002. However, in long-term use of the method in our lab, it was found that the specificity of the method was reduced when common Taq polymerase was employed instead of AmpliTaqGold DNA polymerase (unpublished data). Moreover, Naito's method could not identify subgenotypes directly. Among one round type-specific PCR, Chen's method [12] was reported to be superior in specificity and sensitivity, and could distinguish subgenotypes in another reaction. However, our study showed that the sensitivity of the method for genotypes B and C was >10^4^ IU/mL (the procedures exactly followed those as reported). Other studies also showed similar results about the sensitivity of Chen’s method [23,24].

Considering the importance of genotyping and subgenotyping of HBV in clinical and epidemiology surveys, a more specific, sensitive and low-cost method is required.

This study established a type-specific nPCR assay for genotypes A-D and main subgenotypes of HBV B and C, based on modification of the PCR method developed by Naito *et al.,* (2001) [15], and it was applied to investigate the distribution of HBV genotypes and subgenotypes in China. Using this method, 642 samples from patients with chronic HBV infection from nine provinces in China were assayed.

## Methods

### Samples

Plasmids containing genomic DNA of HBV genotype A [25], genotype D [26], subgenotype B1 [27] and subgenotype C2 [25] were employed in this study. Serum samples of HBV subgenotype B2 and C1, which were confirmed by sequencing, were employed as standard sera [GenBank: JQ416307, JQ416308]. All of the samples were stored at −80 °C before testing.

One hundred and twenty seven serum samples were selected randomly from the serum bank and were used for evaluating the sensitivity of the nPCR method. All of the samples with an HBV DNA load of ≥10^2.3^ IU/mL could be genotyped. A further 543 samples with an HBV DNA load of ≥10^2.3^ IU/mL were then selected from the serum bank. Therefore, a total of 642 clinical serum samples from patients with chronic HBV infection were enrolled in this study.

These serum samples enrolled in this study were collected from the east (Shandong and Jiangsu), the west (Xinjiang), the south (Guangdong and Guangxi), the north (Beijing and Jilin) and the central part (Hebei and Henan) of China. All the samples were obtained from patients with chronic HBV infection. Among the 642 samples, 36 were from Jilin (The First Affiliated Hospital of Jilin University), 163 were from Beijing (Beijing Ditan Hospital), 61 were from Guangdong (Peking University Shenzhen Hospital), 49 were from Guangxi (Guangxi Provincial Center for Disease Control and Prevention), 24 were from Shandong (Shandong Jining Infectious Disease Hospital), 124 were from Jiangsu (Jiangsu Provincial Center for Disease Control and Prevention), 118 were from Xinjiang (The First Affiliated Hospital of Xinjiang Medical University), 32 were from Henan (Zhengzhou Municipal Center for Disease Control and Prevention) and 35 were from Hebei (Handan Municipal Center for Disease Control and Prevention).

Informed consent was obtained from each patient at the time that serum samples were collected for the purpose of HBV testing. The serum samples were transported on dry ice and stored at −80 °C in our lab until analyzed.

The study was approved by the Ethics Committee of Peking University Health Science Center in accordance with the Helsinki Declaration.

### Primer design

For primer design, 150 entire nucleotide sequences of HBV genotypes A-H were retrieved from GenBank, and analyzed by DNAStar software. Primers were then designed in the conserved regions of the HBV genome by Primer Premier 5.0 software. Primer sequences within the same genotype or subgenotype differed by ≤ 2 nucleotides (a strict match at the 3’ end was ensured), while primer sequences differed by ≥3 nucleotides (usually ≥2 nucleotides at the 3’ end) among the different genotypes or subgenotypes. Some primers were modified by nucleotide substitutions in the middle or at the 5’ end of primers to avoid intra- or inter-primer dimer formation.

The first round PCR primers (outer primer pairs) were designed in the regions of partial X-PreC/C genes (primers BF and HBAS-4 V) and partial P-PreS/S genes (primers PF and S4R) for the amplification of all A-D genotypes. In the second round amplification, three reactions were performed flexibly using 3 mixes containing multiple primers. Mix A contained sense primers HB (type B specific), DF (type D specific), C1F (subgenotype C1 specific), C2F (subgenotype C2 specific) and antisense common primers BJA-RV and PR. These primers were used to identify genotypes B, D and subgenotypes C1, C2. Mix B contained sense primers AF1, AF2 and antisense primer BA1R (AF1 and AF2 were degenerate primers, all three primers were type A specific). These primers were used to identify genotype A. Mix C contained sense primer HB (type B specific) and antisense primers BA (subgenotype B2 specific), BJ (subgenotype B1 specific). These primers were used to identify subgenotypes B1 and B2. Genotypes A-D and subgenotypes B1, B2, C1 and C2 could be differentiated based on the length of the PCR products. HBAS-4 V and BJA-RV were designed by Sugauchi *et al*., (2003) [28]. Primer BA1R was designed by Naito *et al*., (2001) [15]. All the primers were synthesized by Shanghai Sangon Biological Engineering Technology & Services, China. The sequences and the positions of the primers were shown in Table 1 and Additional file 1.

**Table 1:** Sequences of primers used in the modified nPCR method

**Primer**	**Sequence**	**Position (nt)**
First PCR		
BF	5’- ACG GGG CGC ACC TCT CTT TA -3’	1 519–1 538
HBAS-4 V	5’- ATA GGG GCA TTT GGT GGT CT -3’	2 316–2 297
PF	5’- TTA TGC CTG CTA GGT TY^a^T ATC C -3’	2 635–2 656
S4R	5’- AGA AGA TGA GGC ATA GCA GC -3’	434-415
Second PCR		
Mix A		
HB	5’- ACC GTG AAC GCC CAC M^b^GG AA -3’	1 617–1 636
BJA-RV	5’- TTC TTT ATA CGG GTC AAT GTC CAT G -3’	1 924–1 900
DF	5’- GCA GAA TCT TTC CAC CAG -3’	2 853–2 870
C1F	5’- TCA CTC CR^c^C CAC ACG GCA A -3’	3 047–3 065
C2F	5’- CAC CGA ACA TGG AGA R^c^CA CA -3’	147-166
PR	5’- TTG GTG AGT GAT TGG AGG TTG -3’	341-321
Mix B		
AF1	5’- GCC TAC TAG ATT CTA TCC TAC CCA C -3’	2 645–2 669
AF2	5’- GCC TAC TAG ATT TTA TCC TAA CAG C -3’	2 645–2 669
BA1R	5’- CTC GCG GAG ATT GAC GAG ATG T -3’	111-132
Mix C		
HB	5’- ACC GTG AAC GCC CAC M^b^GG AA -3’	1 617–1 636
BA	5’- GTG TCG AGR^c^ AGA TCT CGA ATA -3’	1 998–1 978
BJ	5’- TGA TCT TTA GGC CCA TGT TAG T -3’	2 192–2 171

^a^ Y: C or T.

^b^ M: A or C.

^c^ R: A or G.

### DNA extraction and amplification

Hepatitis B virus DNA was extracted from 200 μl serum samples using a QIAamp DNA Blood Kit (Qiagen, Hilden, Germany).

In the first round amplification, the following components were added to each microcentrifuge tube: 2 μl 10× buffer containing 1.5 mM MgCl_2_, 0.4 μl dNTP (10 mM), 1 μl each of primer BF (10 μM) and HBAS-4 V (10 μM), 0.6 μl each primer PF (10 μM) and S4R (10 μM), 1.5 units of *Taq* DNA polymerase (Dongsheng Biotech, Guangzhou, China) and 5 μl template, and then distilled deionized H_2_O (ddH_2_O) was added to make a total reaction volume of 20 μl. The thermocycler (iCycler™; Bio-Rad, Hercules, CA, USA) was programmed to predenature the samples at 95 °C for 5 min followed by 40 cycles: 95 °C for 30 s, 58 °C for 30 s, and 72 °C for 1 min. Final elongation was at 72 °C for 7 min. In the second round of amplification, 3 reactions were performed using the 3 mixes of primers. For the mix A reaction, the following components were added to each microcentrifuge tube: 2 μl 10× buffer containing 1.5 mM MgCl_2_, 0.4 μl dNTP (10 mM), 1 μl each primer (10 μM) of mix A, 1 unit of *Taq* DNA polymerase and 1 μl PCR products of the first round amplification, then ddH_2_O was added to make a total reaction volume of 20 μl. The amplification program was the same as that of the first round PCR except the elongation time was decreased to 45 s. Amplifications with mix B and mix C were exactly the same as those with mix A, except for the primers of each mix. The PCR products of the second amplification, together with molecular weight standards (Takara Bio, Shiga, Japan), were electrophoresed on 2% agarose gels stained with ethidium bromide and examined under UV light using the Bio-Rad Gel Doc 2 000 System (Bio-Rad Laboratories, Segrate, Milan, Italy)**.**

The standard precautions were taken to avoid contamination during PCR, and a negative control was included in each run of tests to ensure the specificity.

### Evaluating the sensitivity of the nPCR method

The quantitation of HBV DNA was determined using the Abbott RealTime HBV DNA assay System, as described previously by Ciotti *et al*., (2008) [29]. HBV DNA was extracted from 200 μl of serum during the sample preparation procedure. The limit of detection was 15 IU/mL (10^1.18^ IU/mL).

The serially diluted plasmids and 127 serum samples, of which the HBV DNA levels were quantitated using the Abbott RealTime HBV DNA Assay System, were used for evaluating the sensitivity of the nPCR method.

### Evaluating the specificity of the nPCR method

First, the plasmids and the standard serum panels were applied for evaluating the specificity of the nPCR method. Six hundred and forty two serum samples were then assayed by the nPCR method. Among them, 130 samples were selected randomly and their PCR products were sequenced directly to confirm the genotypes and subgenotypes detected by the nPCR method (Table 2).

**Table 2:** Samples selected randomly from the 639 clinical samples for sequencing

**Place where serum samples were collected**	**Genotype/Subgenotype**								**Total**
	**B2**	**B1 + B2**	**C1**	**C2**	**D**	**B2 + C2**	**C2 + D**	**B2 + C1 + D**	
Shandong	0	0	1	10	0	2	0	0	13
Guangdong	4	0	3	2	1	3	0	1	14
Guangxi	0	0	0	0	0	4	4	0	8
Henan	0	0	0	0	0	0	1	0	1
Hebei	0	0	0	3	0	0	0	0	3
Xinjiang	0	0	0	4	36	0	3	0	43
Jiangsu	21	1	0	24	0	2	0	0	48
Total	25	1	4	43	37	11	8	1	130

### Evaluating the reproducibility of the nPCR method

The reproducibility of the nPCR method was evaluated using 4 plasmids of genotypes A, B, C and D (10^5^ IU/mL) and 10 clinical samples (including 2 genotype B, 3 genotype C, 3 genotype D and 2 ungenotyped). For each plasmid and sample, the test was repeated ten times.

### DNA sequencing and phylogenic analysis

The primers used for sequencing were as follows: outer primers of PreS/S region PF and S4R; inner primers of PreS/S region SF (nt2817-2839) 5’ TCA CCA TAT ACA TGG GAA CAA GA 3’, and PR3 (nt423-402) 5’ CAT AGC AGC AGG ATG AAG AGG A 3’; outer primers of RT region P5 (nt972-993) 5’-GTG GCT CCA GTT CMG GAA CAG T-3’, and P2 (nt2194-2173) 5’-CTA GGA GTT CCG CAG TAT GGA T-3’; inner primers of RT region P3 (nt976-993) 5’-CTC CAG TTC CGG AAC AGT-3’, and P4 (2170–2153) 5’-GCA GAG GAG CCA CAA AGG-3’. Type-specific primers were adopted to verify the genotypes/subgenotypes in mixed infection samples. DNA sequencing was performed using Applied Biosystems 3730 XL DNA Analyzer.

Phylogenic analysis was carried out using MEGA 4.0 software. The phylogenic trees were constructed by the neighbor-joining method [30]. Bootstrap re-sampling and reconstruction were carried out 1 000 times to confirm the reliability of the phylogenic trees.

## Results

### The sensitivity of the nPCR method

Serially diluted plasmids and clinical samples quantitated using the Abbott RealTime HBV DNA Assay System were used for evaluating the sensitivity of the method.

The genotypes of the plasmids could be identified when the HBV DNA load was ≥10^1.18^ IU/mL.

Of 8 clinical samples with viral loads <10^1.18^ IU/mL, 3 (37.5%) could be genotyped successfully by the nPCR method, while 15 of 38 (39.5%) clinical samples with viral loads of 10^1.18^ IU/mL-10^2.3^ IU/mL could be genotyped successfully. However, 100% of the samples could be genotyped when the viral load was ≥10^2.3^ IU/mL (Table 3).

**Table 3:** Identification of HBV genotypes in clinical samples with different HBV DNA levels

**HBV-DNA level (IU/ml)**	**No. tested**	**No. successfully genotyped**	**Percentage successfully genotyped (%)**	**Genotypes**				
				**A**	**B**	**C**	**D**	**Mix**
<10^1.18^	8	3	37.5	0	0	2	1	0
10^1.18^ -10^2.3^	38	15	39.5	0	0	12	3	0
10^2.3^ - 10^3^	25	25	100	0	1	21	0	1^a^ + 2^c^
10^3^ - 10^4^	12	12	100	0	2	10	0	0
10^4^ -10^5^	4	4	100	0	1	3	0	0
10^5^-10^6^	6	6	100	0	1	5	0	0
10^6^-10^7^	4	4	100	0	1	3	0	0
10^7^-10^8^	12	12	100	0	3	8	0	1^b^
10^8^ -10^9^	18	18	100	0	4	13	0	1^a^
Total	127	99	78.0	0	13	77	4	5

^a^ B and C mixed genotype infections.

^b^ B1 and B2 mixed subgenotype infections.

^c^ C and D mixed genotype infections.

### The specificity of the nPCR method

**Figure 1:**
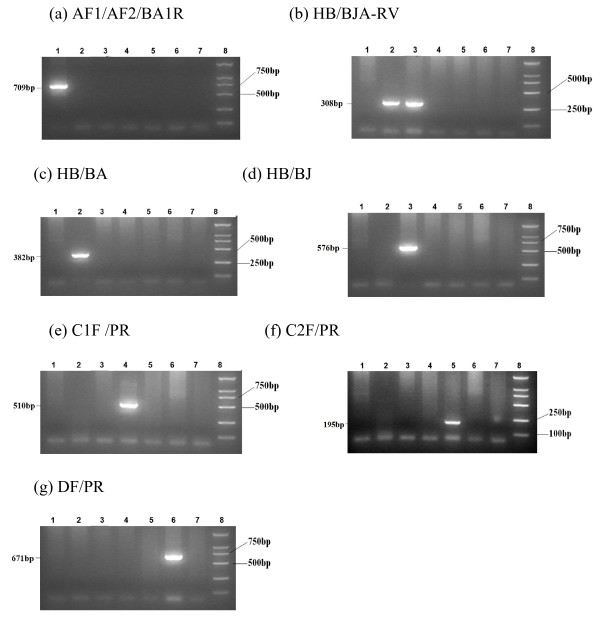
**The specificity of the individual primer pairs was evaluated using plasmids and standard sera containing the HBV genome.** Type-specific primers AF1/AF2/BA1R (Figure 1a) were used to amplify plasmids/standard sera of HBV genotype A (Lane 1), subgenotype B2 (Lane 2), subgenotype B1 (Lane 3), subgenotype C1 (Lane 4), subgenotype C2 (Lane 5) and genotype D (Lane 6). Lane 7 is the negative control, and lane 8 is the molecular weight standard. The PCR product of genotype A was 709 bp. Type-specific primers of genotype B (Figure 1b), D (Figure 1g) and subgenotype B2 (Figure 1c), B1 (Figure 1d), C1 (Figure 1e) and C2 (Figure 1f) were also used to amplify from these samples. The PCR products of genotype B, subgenotype B2, B1, C1, C2 and genotype D were 308 bp, 382 bp, 576 bp, 510 bp, 195 bp and 671 bp, respectively.

**Figure 2:**
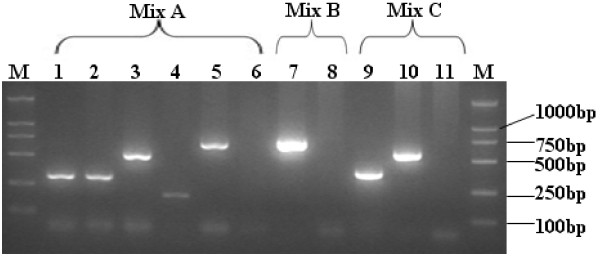
**The specificity of the 3 multiplex mixes of primers (A, B and C) evaluated using plasmids and standard sera containing the HBV genome.** M: molecular weight standards; Lane 6, 8 and 11: negative controls; Lane1 and 9: standard sera of subgenotype B2; Lane 2 and 10: plasmid of subgenotype B1; Lane 3: standard serum of subgenotype C1; Lane 4: plasmid of subgenotype C2; Lane 5: plasmid of genotype D; Lane 7: plasmid of genotype A.

The specificity of the primer pairs was evaluated using the plasmids and standard serum panels as mentioned above. As shown in Figures 1 and 2, the individual primer pairs and three multiplex mixes of primers (mix A, mix B and mix C) exclusively amplified HBV of the respective genotype/subgenotype, and no amplification of other HBV genotypes/subgenotypes was observed.

Of the 642 samples tested, 639 (99.5%) were genotyped and subgenotyped successfully. To confirm the results detected by the nPCR method, 130 samples were selected randomly from the 639 clinical samples as shown in Table 2 (109 single-genotype infection samples, one single-genotype infection sample coexistence of different subgenotypes and 20 samples coexistence of different genotypes), and their PCR products were sequenced directly [GenBank: JQ416163-JQ416306, HQ833465-HQ833471]. All the sequencing results were consistent with those of the nPCR method.

### The reproducibility of the nPCR method

For 4 plasmids of genotypes A, B, C and D (10^5^ IU/mL) and 10 clinical samples (including 2 genotype B, 3 genotype C, 3 genotype D and 2 ungenotyped), consistent genotyping results were obtained from ten independent experiments. The ungenotyped samples could not be genotyped in these ten independent experiments.

### Geographical distribution of HBV genotypes and subgenotypes

Among the 642 serum samples studied, the distribution of HBV genotypes was as follows: genotype B: 72 (11.2%); genotype C: 438 (68.2%); genotype D: 46 (7.2%) and 84 (13.1%) patients were mixed genotypes. There was no other genotype found in this study. The sequencing analysis of HBV DNA from the 20 samples with mixed HBV genotypes confirmed the results of nPCR methods.

**Figure 3:**
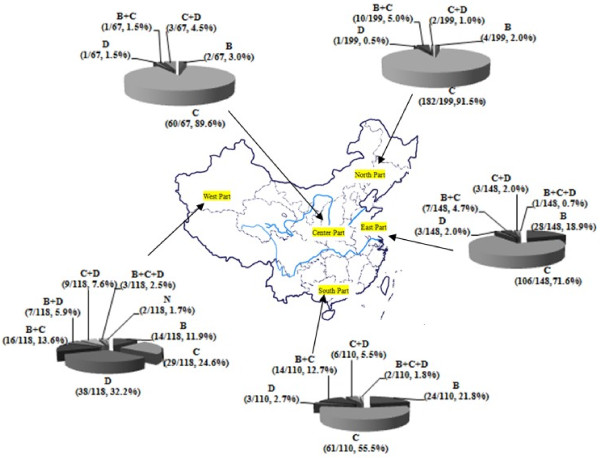
**Distribution of HBV genotypes among different areas of China detected by the nPCR method (n = 642).** M, mixed genotype infection; N, the strains that were not genotyped by the nPCR method.

Geographically, the predominant genotype in China was genotype C (Figure 3 and Additional file 2), accounting for 91.5% (182/199) in the north, 89.6% (60/67) in the central area, 71.6% (106/148) in the east, 55.5% (61/110) in the south and 24.6% (29/118) in the west of China. Genotype B was mainly found in the south (21.8%, 24/110), the east (18.9%, 28/148) and the west (11.9%, 29/118) of China, while it was only 2.0% in the north and 3.0% in central China. Genotype D was prevalent in the west (Xinjiang Uygur Autonomous Region). As for mixed genotype infection, B + C was predominant and could be detected in all part of China.

The major subgenotypes were B2 (87.5%, 63/72) among genotype B, and C2 (92.9%, 407/438) of genotype C (Table 4), and they were the predominant subgenotype in all part of China as well. Subgenotype C1 was mainly found in the south of China. Subgenotype B1 (0.6%, 4/642) was found in Xinjiang. Mixed subgenotypes B1 + B2 (0.6%, 4/642) and C1 + C2 (0.9%, 6/642) were sporadically distributed in the east, the west and the south of China, and had rarely been reported before.

**Table 4:** Distribution of HBV subgenotype B and C in different areas of China

	**Sub-genotype B% (m/n)**				**Sub-genotype C% (m/n)**		
	**B1**	**B2**	**B1 + B2**	**B**^**N**^	**C1**	**C2**	**C1 + C2**
East		96.4 (27/28)	3.6 (1/28)		0.9 (1/106)	99.1 (105/106)	
West	28.6 (4/14)	42.9 (6/14)	21.4 (3/14)	7.1 (1/14)		93.1 (27/29)	6.9 (2/29)
South		100 (24/24)			39.3 (24/61)	54.1 (33/61)	6.6 (4/61)
North		100 (4/4)				100 (182/182)	
Center		100 (2/2)				100 (60/60)	
Total	5.6 (4/72)	87.5 (63/72)	5.6 (4/72)	1.4 (1/72)	5.7 (25/438)	92.9 (407/438)	1.4 (6/438)

N: the strain that could not be subgenotyped by nPCR.

## Discussions

A relatively simple, rapid, economic and practical genotyping system with high sensitivity and specificity was established based on the methods developed by Naito *et al.,* (2001) [15] and Sugauchi *et al.,* (2003) [28]. Compared with the classical type-specific PCR method established by Naito *et al.,* (2001) [15], low-cost common Taq polymerase was used in our method. The cost of the common Taq polymerase is roughly 40% of AmpliTaq Gold DNA polymerase used in Naito’s method. When common Taq polymerase was employed instead of AmpliTaq Gold DNA polymerase in Naito’s method, the specificity of the method was reduced (unpublished data). Only a single nucleotide difference between type-specific primers (e.g. antisense primer BB1R) and reference sequences of different genotypes may allow extension of mismatched primer-template. It was reported that, in some instances, a single terminal 3'-mismatched base did allow PCR amplification to proceed [31,32], which reduced the specificity of type-specific PCR method. In our method, primers specific for each HBV genotype or subgenotype differed by ≥3 nucleotides (≥2 nucleotides at the 3’ end of the sequences). Double mismatches at 3’ end decreased the extension of mismatched primer-template. The sequencing results of 130 serum samples selected randomly from the 639 samples, confirmed the specificity of our nPCR method.

The sensitivity of the nPCR method was evaluated using serially diluted plasmids (10^1^-10^9^ IU/mL) and 127 clinical samples, in which HBV DNA was quantitated using the Abbott RealTime HBV DNA Assay System. The limit of detection was <10^1.18^ IU/mL (Table 3) and 100% of the samples could be genotyped when the viral load was ≥10^2.3^ IU/mL, which was sufficient for routine genotyping and subgenotyping. The sensitivity of Chen’s method [12] using single-round PCR for detection of genotype B and C was >10^4^ IU/mL as we repeated this method in our lab. Therefore, we developed a nested PCR method for HBV genotyping and subgenotyping to improve the lower limit of single-round PCR method.

On the other hand, there were two fragments generated after the first round PCR reaction in the nPCR method. One of the fragments, for the specific detection of genotype B, was located mainly in the preC/C region of the HBV genome, and another one, for detecting genotypes C and D, was mainly in the preS/S region (see Supplementary data 1). This design increased the sensitivity and specificity of the detection of genotypes B, C and D, while mixed strains could be detected more sensitively as well. In this study, 13.1% (84/642) of the samples assayed by the nPCR method were mixed genotypes. The sequencing analysis of HBV DNA from the 21 cases with mixed HBV infection confirmed the results of the nPCR method (random sampling cases were 1/5 of total number of mixed infection samples).

Only three of the 642 samples could not be genotyped or subgenotyped by the nPCR method. Two of the three samples could not be genotyped and another one was identified as genotype B but could not be subgenotyped further by the nPCR method. The sequencing results of the three samples revealed that the first 2 samples were C/D recombinants. It was reported that this kind of C/D hybrid emerged in the preS/S region between genotype D and subgenotype C2, and belonged to genotype C. The recombination fragment of genotype D was located at the nt 10–799 or nt 10–1 499 region [33]. As primers DF and C2F were designed at nt2 853–2 870 and nt147-166 respectively, the nPCR method could not detect this kind of C/D recombinant. Until now, whole genome sequencing was the only effective method to detect C/D recombinants. The third sample, which was identified as subgenotype B2 by sequencing, had a mutation in the region where primer BA was designed, and the mutation was exactly at the nucleotide position matching the 3’ end of the primer, which led to failure of detection by the nPCR method. However, the frequency of this kind of mutation was relatively low.

The present study demonstrated that genotype B, C and D were circulating in China. The results revealed marked geographical differences in the distribution of HBV genotypes. In the north and central China, genotype C was the predominant while the percentage of other genotypes was low. In the south and the east of China, the dominant genotypes were C and B. In the west, the dominant genotype was D. It was obvious that the percentage of mixed genotype infection was high in the south and the west of China. As previously reported, HBV infection rate with multiple genotypes was found to be 31% in Shenzhen [34] (in the south of China), and 22%- 43.8% in Xinjiang [35,36] (in the west of China). In China, studies on the subgenotype of HBV were seldom reported. The predominant subgenotype was B2 and C2 in all parts of China.

## Conclusions

In conclusion, an accurate, sensitive and cost-effective nPCR genotyping method for the simultaneous detection of the HBV genotypes A to D and subgenotypes B and C has been established. The geographical distribution of HBV genotypes and subgenotypes in the mainland of China was mapped at the same time. Further investigation of HBV genotypes and subgenotypes using this method will enrich the epidemiological data for HBV in regions where genotypes A-D are predominant. This method will provide a useful tool for clinical and epidemiological applications.

## Abbreviations

ddH2O: Distilled deionized H2O; HBsAg: Hepatitis B surface antigen; HBV: Hepatitis B virus; nPCR: Nested polymerase chain reaction; PreC: Pre Core; PreS: Pre Surface; RFLP: Restriction fragment length polymorphism; RT: Reverse transcriptase.

## Competing interests

The authors declare that they have no competing interests.

## Authors’ contributions

JL, HZ, JW, HJ designed this study; JJN, KXS, LY, JW, HJ, JXY, MSS performed lab work; JJN and KXS participated in data analysis. JJN and JL drafted the manuscript. HZ, JL, LW, FML, TL critically reviewed the manuscript. All the authors read and approved the final manuscript.

## Supplementary Material

Additional file 1 Figure S1: The positions of the type-specific primers for the improved nPCR method. Click here for file

Additional file 2 Table S1: The genotype distribution in nine provinces located in different areas of China. Click here for file
