# Supplementary material for: A type-specific nested PCR assay established and applied for investigation of HBV genotype and subgenotype in Chinese patients with chronic HBV infection
Source: Virol J. 2012 Jun 19;9:121. doi: 10.1186/1743-422X-9-121 (PMC3477104; doi:10.1186/1743-422X-9-121)
Supplement: Additional file 1 — Figure S1: The positions of the type-specific primers for the improved nPCR method. [file 1743-422X-9-121-S1.doc]

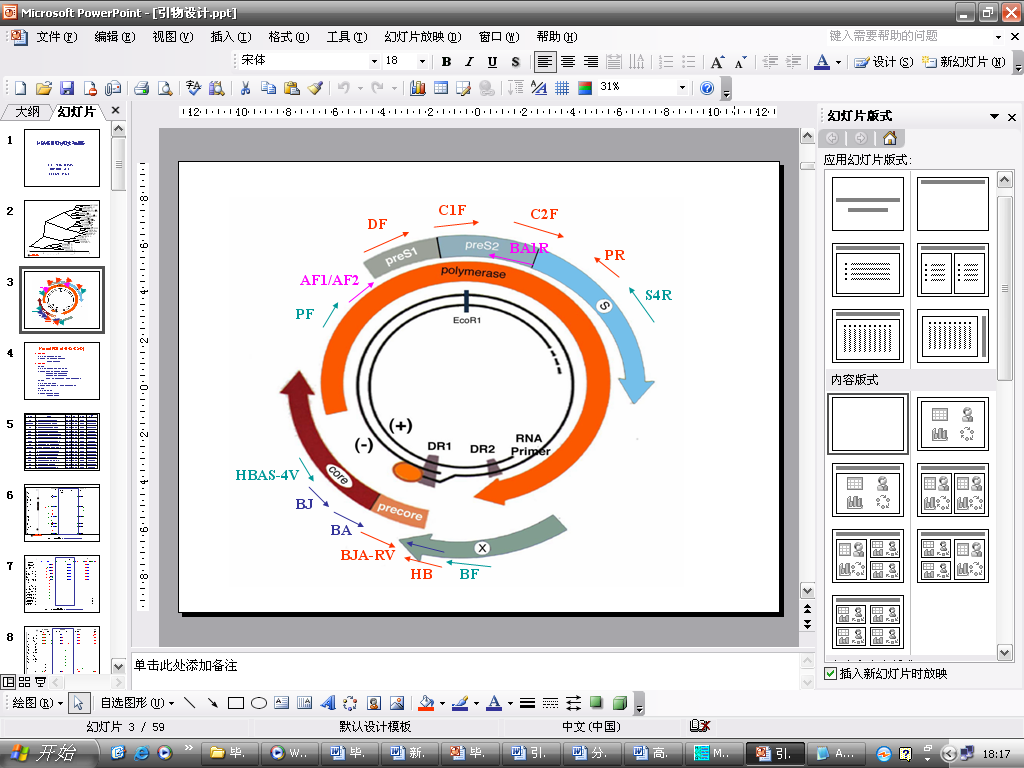


**Additional file 1 The positions of the type-specific primers for the improved nPCR method.** The exact locations of the primers were shown in Table 1.
