# Supplementary material for: A type-specific nested PCR assay established and applied for investigation of HBV genotype and subgenotype in Chinese patients with chronic HBV infection
Source: Virol J. 2012 Jun 19;9:121. doi: 10.1186/1743-422X-9-121 (PMC3477104; doi:10.1186/1743-422X-9-121)
Supplement: Additional file 2 — Table S1: The genotype distribution in nine provinces located in different areas of China. [file 1743-422X-9-121-S2.doc]

**Additional file 2 The genotype distribution in nine** provinces located in different areas of China

|  | B | C | D | B+C | B+D | C+D | B+C+D | N | Total |
| --- | --- | --- | --- | --- | --- | --- | --- | --- | --- |
| **East** |  |  |  |  |  |  |  |  |  |
| Shandong | 0 | 16 | 0 | 4 | 0 | 3 | 1 | 0 | 24 |
| Jiangsu | 28 | 90 | 3 | 3 | 0 | 0 | 0 | 0 | 124 |
| **West** |  |  |  |  |  |  |  |  |  |
| Xinjiang | 14 | 29 | 38 | 16 | 7 | 9 | 3 | 2 | 118 |
| **South** |  |  |  |  |  |  |  |  |  |
| Guangdong | 18 | 28 | 1 | 10 | 0 | 2 | 2 | 0 | 61 |
| Guangxi | 6 | 33 | 2 | 4 | 0 | 4 | 0 | 0 | 49 |
| **North** |  |  |  |  |  |  |  |  |  |
| Beijing | 3 | 152 | 0 | 8 | 0 | 0 | 0 | 0 | 163 |
| Jilin | 1 | 30 | 1 | 2 | 0 | 2 | 0 | 0 | 36 |
| **Center** |  |  |  |  |  |  |  |  |  |
| Hebei | 1 | 33 | 0 | 0 | 0 | 1 | 0 | 0 | 35 |
| Henan | 1 | 27 | 1 | 1 | 0 | 2 | 0 | 0 | 32 |
| **Total** | 72 | 438 | 46 | 48 | 7 | 23 | 6 | 2 | 642 |

N: the strain which couldn’t be genotyped by nPCR
